# Biomathematical Description of Synthetic Peptide Libraries

**DOI:** 10.1371/journal.pone.0129200

**Published:** 2015-06-04

**Authors:** Timo Sieber, Eric Hare, Heike Hofmann, Martin Trepel

**Affiliations:** 1 Department of Oncology and Hematology, University Medical Center Hamburg-Eppendorf, Hamburg, Germany; 2 Department of Statistics, Iowa State University, Ames, IA, USA; 3 Department of Hematology and Oncology, Augsburg Medical Center, Interdisciplinary Cancer Center, Augsburg, Germany; Imperial College London, UNITED KINGDOM

## Abstract

Libraries of randomised peptides displayed on phages or viral particles are essential tools in a wide spectrum of applications. However, there is only limited understanding of a library's fundamental dynamics and the influences of encoding schemes and sizes on their quality. Numeric properties of libraries, such as the expected number of different peptides and the library's coverage, have long been in use as measures of a library's quality. Here, we present a graphical framework of these measures together with a library's relative efficiency to help to describe libraries in enough detail for researchers to plan new experiments in a more informed manner. In particular, these values allow us to answer-in a probabilistic fashion-the question of whether a specific library does indeed contain one of the "best" possible peptides. The framework is implemented in a web-interface based on two packages, discreteRV and peptider, to the statistical software environment R. We further provide a user-friendly web-interface called PeLiCa (*Pe*ptide *Li*brary *Ca*lculator, http://www.pelica.org), allowing scientists to plan and analyse their peptide libraries.

## Introduction

Since the year 2000 we see on average more than 500 publications a year that are based on the use of peptide libraries (PubMed query April 2014 on “peptide library”). This serves as a good measure to reflect on the importance of peptide libraries in a wide spectrum of biological applications ranging from the identification of protein interaction sites (e.g. [1]) and the development of enzyme inhibitors (e.g. [2]) to identification of peptides that mediate cell type specific gene delivery by viral vector systems (e.g. [3]). In all of these applications, chemically synthesized random oligonucleotides are introduced into plasmids encoding structural proteins of bacteriophages [4] or viruses, such as adeno-associated viruses [3], adenoviruses [5] or retroviruses [6]. Plasmids are then ligated and transformed into bacteria to generate a plasmid library, which in turn is used to produce virus or phage libraries. These can be utilised in a variety of selection procedures, aiming to isolate peptide bearing viruses and phages with desired properties or scaffold independent, functional peptides (e.g. peptide inhibitors [2]). The success of this method is highly dependent on the diversity of the initial pool of peptides, as the chance to identify the “best possible” sequence, or even a suitable sequence, is directly correlated with the number and diversity of the peptides in the library used for the screening procedure. A cheap, simple and powerful way to investigate, if the production of a library was successful is the Quick-Quality-Control (QQC) [7, 8]. In short, library material is pooled and used in a single sanger sequencing run to uncover undesired imbalances in the ratios of inserted bases as well as production errors like primer-dimer insertions etc., which might lead to a reduced library diversity.

Determining the diversity of a library is problematic, though, as the number of distinct peptides, which we will refer to as *peptide diversity*, cannot be measured easily. Direct measurements are generally impracticable: even though next-generation sequencing is now widely accessible, the sheer size of current libraries (e.g. 2 × 10^10^ clones [9]) makes the use of this technique for counting purposes prohibitive due to the time and financial effort associated with the very high sequencing depth required for a sufficient sequencing coverage. Other approaches of measuring library diversity in the literature include DeGraaf et al. [10], who estimate diversity of their phage decapeptide display library from the distribution of single amino acids and dipeptides in a sample. Rodi et al. define *functional diversity* as a measure of the distribution of peptides encoded in the library [11, 12]. Both methods, functional diversity and peptide diversity, give valuable distributional information about peptide libraries. A library with an even distribution of sequence frequencies is advantageous, as all peptides enter the selection process in comparable numbers. This supports a swift and successful selection of a suitable peptide. However, peptides that match the selection criteria can be gradually enriched during the selection process, even if they are vastly underrepresented in the initial library. A limitation of functional diversity is that it is a theoretical measure based purely on the library scheme. Functional diversity therefore does not represent the actual number of distinct peptides in a library, which increases with growing size independently of its scheme.

Therefore, many researchers estimate diversity at the level of the plasmid library by counting successfully transformed bacterial colonies (e.g. [13–15]). This number is easily assessable, and represents the maximally achievable diversity for the phage/virus library, as the diversity cannot be increased after the cloning and transformation process. Particular precautions must be taken to avoid—or at least, to minimise—losses to diversity in all steps of the library production to make the number of bacterial colonies a valid qualifier for the peptide library [16]. The number of bacterial colonies on its own is of limited value, as the relevant metric is the number of distinct peptides in the library. However, the two measures are correlated and the number of bacterial colonies can be used to estimate peptide diversity. Peptide diversity of the library is always lower than colony number, due to the possibility that different bacterial clones encode identical peptides. This is caused by several clones containing identical peptide encoding DNA and/or by clones harboring distinct DNA sequences that encode the same peptide due to the degenerate nature of the genetic code: amino acids are encoded by up to six distinct codons; multiple DNA sequences can therefore describe the same peptide. This has the effect that, for instance, a pool of randomised codon DNA sequences of length seven has a nominal diversity of 64^7^ (64 codons; 4.4 × 10^12^) while it encodes only 23^7^ (20 amino acids and three stop codons; 3.4 × 10^9^) distinct amino acid sequences. Further, stop codons in the random nucleotide sequence prematurely terminate the peptide and can cause dysfunctional proteins in display systems [17, 18]. Libraries are therefore often encoded by limited subsets of the standard 64 codons to at least partially counteract both effects (as also discussed in [19]). Instead of the NNN scheme, where “N” represents any of the four bases, encoding schemes like NNB, NNK or NNS (B: C/G/T; K: G/T; S: G/C) are used. These schemes encode all twenty amino acids and one stop codon each, while the total number of codons is reduced to 48 (NNB) and 32 (NNK and NNS), respectively. Apart from the mentioned, a number of further encoding schemes exist. These are primarily developed in the framework of saturation mutagenesis, another area in which randomisation libraries are used. Special attention in saturation mutagenesis received the MAX randomization [20], the 22c trick [21] and the “small-intelligent libraries” [22]. However, as these techniques are not suited to produce long stretches (i.e. five or more amino acid positions, [23]) of randomized sequences, they are not used for the production of peptide libraries.

One approach to overcome the problematic stemming from the degenerate nature of the genetic code is common to both peptide libraries and saturation mutagenesis and consists of libraries in which the ratio of the number of codons for each amino acid is one. From here on, we will refer to these libraries as 20/20 libraries (20 codons for 20 amino acids). 20/20 libraries also allow a complete avoidance of stop codons, which have been shown to increase functional diversity in phage display [24].

The most common method to produce such peptide libraries is the trimer approach. In trimer libraries [25] oligonucleotides are synthesised by assembling pre-fabricated trinucleotide phosphoramidites or trimers. An alternative to the trimer approach to generate 20/20 libraries is the ProxiMAX system [23].

Another important consideration regarding peptide diversity are cysteines. Pairs of cysteines flanking randomised sequences are often used in phage display as they form controlled disulfide bridges that enhance half-lives and binding characteristics of the library peptides [26]. However, random integration of odd numbers of cysteines has repeatedly been shown to inhibit the generation of peptide bearing phages [27]. Further, even though the situation is less well understood for other display systems, a strong underrepresentation of cysteine-containing peptides was observed in peptide libraries on different adeno-associated virus (AAV) vectors [28–31]. This again suggests unfavorable effects of cysteine incorporation on basic functions of the display system. In line with this is the notable lack of capsid surface-exposed cysteine residues on wild type AAV2 [32]. Also, the surface of human adenovirus type 5 is naturally devoid of cysteines. If they are artificially integrated, the particles were shown to be prone to aggregation due to the formation of interparticle disulfide bridges [33].

With regard to the aforementioned factors, we will determine peptide diversity by using the number of bacterial clones, but consider effects of encoding schemes and stop codons. For the purpose of discussing diversity, we will regard cysteine-containing peptides as non-functional unless otherwise mentioned. A complete discussion of diversity of libraries treating cysteines as valid or invalid can be found at our website PeLiCa (available at http://www.pelica.org). Other biological restraints that negatively affect peptide diversity do exist, but are not taken into account here, as they are largely unknown and highly dependent on the individual system and its specific characteristics, such as the differences between distinct incorporation sites [29, 34]. However, depending on the system and its intended use (e.g. generation of a functional viral vector with peptide mediated tropism), compatibility with such restrictions might be considered as a first step in the selection process.

Determining the peptide diversity is a mathematically taxing problem that becomes ever more challenging with increasing peptide length. In particular, Monte Carlo simulation is not practical for this purpose. There are two primary limitations:

1. For library sizes above about 10^8^, the speed of the simulation even on modern hardware is prohibitive without the use of massively parallel hardware.
2. Small probabilities (such as we deal with for rare peptides in a library) cannot be accurately estimated by Monte Carlo methods without oversampling. Oversampling does further increase the complexity of the simulation by increasing the number of runs that need to be made.

In this publication, we revisit the mathematical framework capable of facilitating this task, drawing from different sources [35–37]; the quality of a peptide library is not only defined by the peptide diversity, we further use the concepts of expected coverage, relative efficiency to allow a more detailed evaluation of libraries. Further, we discuss effects of insert length, different encoding schemes (NNN, NNB, NNK, NNS, and 20/20), and in particular answer one of the important questions for researchers working with peptide libraries: “What are the chances that my library contains (one of) the ‘best’ possible peptides?”

Our framework allows to determine the peptide diversity of large peptide libraries by combining quantitative information about the number of clones with qualitative information about biological, statistical and encoding effects. This in turn facilitates a deeper understanding and allows for a more informed planning of new, optimized libraries. To make the framework easily accessible, we generated a user-friendly web-interface called PeLiCa, which allows the user to determine all of these factors for libraries of sizes up to 9.9 × 10^25^ bacterial clones, using different encoding schemes (including custom-designed schemes and those that consider cysteine viable) and peptide lengths. PeLiCa is implemented in a web-interface based on two packages, discreteRV [38, 39] and peptider, [40], to the statistical software environment R [41].

## Methods

### Measuring Diversity

While not studied in detail for peptide libraries, studies on diversity at the amino acid level have been performed in the related field of site saturation mutagenesis generated protein libraries. Here, proteins are mutated at a limited number of positions to detect variants with improved properties. The GLUE-IT software (available at http://guinevere.otago.ac.nz/stats.html [37]) generates values for diversity and coverage for protein libraries with up to six modified codons per protein. GLUE-IT was designed for another purpose and does not allow evaluation of cysteines as disruptive, but it can also be used to gain some information for peptide libraries with short peptides. However, it is no longer sufficient to describe most libraries currently used, which are generally longer and range from five up to twenty or more amino acids in length (e.g. [27, 29, 42]).

In our approach to develop a mathematical framework we consider only peptide libraries that are based on synthetic randomized oligonucleiotides. This asserts, from a statistical point of view, that all DNA sequences inserted into the library plasmids are completely randomised and can be observed multiple times.

We discuss three measures of library quality: *peptide diversity* defined—as stated before—as the number of distinct peptides in a library, *expected coverage*, describing the expected fraction of all theoretically possible peptide sequences covered by the library, and *relative efficiency* given as the ratio of the expected number of distinct peptides in a library relative to the overall number of encoding oligonucleotides. The terms *diversity* and *completeness* used by Firth and Patrick [37] for saturation mutagenesis experiments are equivalent to the concepts peptide diversity and expected coverage, we use here for peptide libraries.

We investigate these measures for a set of different encoding schemes: NNN-C, NNK/S-C, NNB-C, and 20/20-C. The -C indicates that we will exclude cysteines from consideration.

Note that the 20/20-C notation refers to libraries that are composed of only the 19 valid amino acid codons and do not include the codon for cystein or any of the stop codons. We will first discuss library properties for libraries with equal codon representation, such as we see in 20/20 libraries, and then extend the situation to other library schemes.

### Libraries with equal codon representations

An easily tractable case for determining diversity is the setting in which all sequences have the same probability of being included in the library. This can be assumed if diversity is investigated at DNA level or for the special case of 20/20 libraries in which every amino acid is represented by one codon. In that case, calculating expected peptide diversity of a library is relatively simple: the probability that a peptide is present in the library is determined by the maximum number of different peptide sequences and the size of the library (note that this is also true, when each amino acid is represented by the same number of codons). Denote the number of all different possible peptides in the library by b, the size, measured as the number of bacterial colonies, of the library by N.

Let us denote the *diversity of this library*, as measured by the number of different peptides, as Z = Z_N,b_. The number of different peptides, Z, that can actually be achieved in the library is the primary point of interest. In practice, the value of Z will differ from library to library, but we can determine an expected value of library diversity, E[Z], and its corresponding variance Var[Z] as outlined below (see also [43]).

**Theorem 1.** For a library of size N chosen from a scheme with b different peptides, which are assumed to be all equally likely, the expected value and the variance of the number of different peptides Z_N,b_ in the library is given as:

$$\begin{array}{ccc} E[Z_{N,b}] & = & b(1-{\left(1-b^{-1}\right)}^{N})\approx b\left(1-e^{-N/b}\right). \end{array}$$ (1)

$$\begin{array}{ccc} \text{Var}[Z_{N,b}] & = & b[{\left(1-b^{-1}\right)}^{N}-{\left(1-2b^{-1}\right)}^{N}]-b^{2}[{\left(1-b^{-1}\right)}^{2N}-{\left(1-2b^{-1}\right)}^{N}]\\ & \approx & b\left(e^{-N/b}-e^{-2N/b}\right)-Ne^{-2(N-1)/b}. \end{array}$$ (2)

The approximation becomes more accurate as the values of b and N increase. For values of b and N above 50 the approximation is already correct to within 1% of the exact value. The relative standard deviation, or the square root of the variance divided by the mean, is negligibly small for most libraries. The proof and a more detailed discussion of the approximation error can be found in S1 and S2 Texts.

In investigating DNA diversity in site saturation mutagenesis libraries, other groups [35, 36] obtained the same result for expected diversity as Theorem 1 based on a Poisson approximation. While this approach is usable for an analysis at the DNA level or 20/20 libraries, it cannot be used directly for library schemes in which the number of codons per amino acid varies, because in this case, the probability that a peptide will be included in the library depends on the sequence. In a standard 64 codon based library there are one to six codons describing individual amino acids (aa). Therefore, some peptide sequences like SLRLLRS are encoded by 6^7^ = 279,936 distinct codon sequences, as each amino acid in the sequence has six independent possibilities to be encoded. At the other end of the scale, there are peptides that are encoded by only a single nucleotide sequence. We will therefore partition the overall library into classes of peptides that all have the same number of encodings (similar conceptual approaches have previously been mentioned, e.g. [37, 44]) and determine overall diversity based on diversity seen within each of these classes. For that, we need to specify the library under observation in more detail.

### Partitioning of Peptide Libraries

To be able to determine the peptide diversity, we have to partition the libraries. In the following, we focus on the 32 codon-based encoding schemes NNK and NNS. Other schemes work similarly, see the class partitioning of NNN-C (S1 Table) and NNB-C (S2 Table). According to the degree of codon redundancy and functionality NNK and NNS are equivalent, and we can distinguish four classes of aa based on a modified NNK/S scheme, in which cysteine is excluded from the set of valid amino acids (Table 1). Amino acids are given in single letter code. Size s defines the number of different amino acids in an aa class, the number of codons, c, reflects how many codons describe each amino acid in the class. Classes A to C contain all codons for feasible amino acids, while class Z contains corruptive codons. The number of valid aa classes is therefore 3. Stop codons as well as cysteines are treated as non-viable amino acids (aa class ‘Z’); sequences containing one or more of these codons will therefore be excluded.

**Table 1:** NNK/S-C Library Scheme.

**aa class**	**amino acids**	**size s**	**# codons c**
A	S, L, R	3	3
B	A, G, P, T, V	5	2
C	D, E, F, H, I, K, M, N, Q, W, Y	11	1
Z	cysteine C, stop TAG	2	1

We are now employing a two-step analysis to retrieve all the relevant probabilistic information to calculate peptide diversity in the resulting library: In a first step we are only interested in whether the outcome is a *valid sequence*, defined to be the case when there is no element of aa class Z in the sequence. Valid sequences are therefore those that are expected to be functional in the biological system. In a second step we will investigate the diversity among the remaining peptide sequences.

Any peptide sequence containing a member of aa class Z is by definition not useful for further analysis. In a randomly generated NNK/S-C library of heptapeptides, these make up 36.35% = 1−(1−*P*(*Z*))^7^ of the total. We will call this percentage of invalid sequences the *initial loss*, L, and restrict our analysis to valid sequences only.

Analysing peptide sequences directly is too computationally complex of a problem. In order to reduce this complexity, we only differentiate between peptide sequences at the level of the previously introduced classes. Let V represent the total number of valid aa classes in the given encoding scheme. Then V^k^ is the total number of peptide classes in a library with peptides of length k. If this is performed for an exemplary library of dipeptide sequences, we have a set of nine different peptide classes as shown in Table 2. The peptide class (first line) is defined by the aa class memberships of their codons as defined for NNK/S-C libraries in Table 1. The number of different unique peptide sequences in each class (second line), and the number of codon representations for each peptide sequence in the class (third line) are given. Within each of the V^k^ = 9 peptide classes, all peptides have an equal number of oligonucleotide sequence representations. This compares to the 19^2^ = 361 possibilities that must be taken into account without the use of peptide classes in the dipeptide case.

**Table 2:** All NNK/S-C peptide sequences of length two partitioned according to peptide classes.

**peptide class**	**AA**	**AB**	**AC**	**BA**	**BB**	**BC**	**CA**	**CB**	**CC**
*# peptides*	9	15	33	15	25	55	33	55	121
*# oligonucleotides*	9	6	3	6	4	2	3	2	1

The peptide class completely determines both the number of unique peptides and the number of nucleotide representations for each of the peptide sequences. For a given sequence, let s_A_, s_B_, and s_C_ represent the number of different amino acids in aa classes A, B, and C, and c_A_, c_B_, and c_C_ stand for the number of codons per amino acid within the corresponding aa class. Here, n_A_, n_B_, and n_C_ refer to the number of elements from each of the aa classes A, B, and C that make up the peptide sequence. The sum of n_A_, n_B_, and n_C_ then adds up to the total length of the sequence.

The number of peptides (# peptides) and corresponding nucleotide representations for each peptide (# oligonucleotides) is then calculated as

$$\begin{array}{ccc} \text{\# peptides} & = & {s_{A}}^{n_{A}}\cdot {s_{B}}^{n_{B}}\cdot {s_{C}}^{n_{C}}.\\ \text{\# oligonucleotides} & = & {c_{A}}^{n_{A}}\cdot {c_{B}}^{n_{B}}\cdot {c_{C}}^{n_{C}}. \end{array}$$

The number of oligonucleotide sequences representing a whole peptide class is given as the product of the number of peptides and the number of individual codon representations per peptide. Under the assumption that in a library of peptides with a length of k amino acids all viable codons v (30 codons for NNK/S-C usage, excluding any class Z codons) are represented with the same probability, this allows us to calculate the probability p for a peptide class to be present in a library as

$$\begin{array}{c} p=\text{\# peptides}\cdot \text{\# oligonucleotides}/v^{k}. \end{array}$$ (3)

### Diversity in general peptide libraries

Combining the information from individual peptide classes we can determine the diversity in the general peptide library.

For a k-peptide library of size N we expect Np_i_ sequences to be selected from peptide class i, where p_i_ is the probability (effectively, the size) of peptide class i. Within this class, all peptides are represented by the same number of oligonucleotide sequences. Assuming b_i_ different peptides in peptide class i are theoretically possible, we have, according to theorem 1, an expected diversity given by the number of different peptides as $b_{i}(1-e^{-Np_{i}/b_{i}})$, resulting in an overall expected number of different peptides in the library and associated variance of

$$\begin{array}{ccc} D(N,k) & = & \sum_{i=1}^{V^{k}}b_{i}\left(1-e^{-Np_{i}/b_{i}}\right). \end{array}$$ (4)

$$\begin{array}{ccc} \sigma_{D}^{2} & = & NL\left(1-L\right)+\sum_{i=1}^{V^{k}}\sigma^{2}\left(Z_{Np_{i},b_{i}}\right), \end{array}$$ (5)

where L is the initial loss of the library scheme for peptides of length k. A simulation-based discussion of this result and the precision of its approximation can be found in S3 Text.

## Results

### Expected coverage and relative efficiency

Based on the overall peptide diversity, we now define two indices measuring different aspects of quality of k-peptide libraries: expected coverage and relative efficiency.

**Definition 1** (Expected coverage). For a k-peptide library of size N the expected coverage and associated variance is defined as

$$\begin{array}{ccc} C(N,k) & = & D\left(N,k\right)/19^{k}.\\ \sigma_{C}^{2} & = & \sigma_{D}^{2}/19^{2k}. \end{array}$$

Expected coverage is an index in [0, 1]. 0 indicates that no peptide is in the library (which can only happen for a library of size 0), and 1 indicates that every single possible peptide is included in the library.

**Fig 1:**
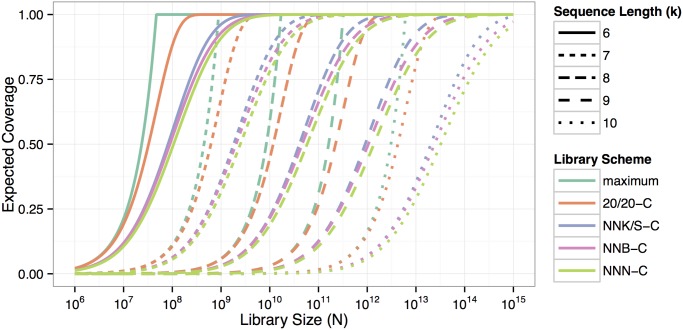
Overview of expected coverage for k-peptide libraries of different sizes N with the different encoding schemes (NNN-C, NNB-C, NNK/S-C, and 20/20-C). The ‘maximum’ line represents the best-case scenario of coverage of a library, in which no peptide appears twice (until the upper limit of all possible peptides is reached at b = 19^k^, at which point duplication is unavoidable).

Fig 1 shows the expected coverage of k-peptide libraries of sizes between 10^6^ and 10^15^ with different encoding schemes. It is obvious that increasing peptide length k has a dramatic negative influence on the expected coverage for a given library size N. Additionally, the used encoding scheme has a profound effect on expected coverage, with 20/20-C libraries being far superior to the other schemes (see also [16, 21, 45, 46]). The line corresponding to ‘maximum’ represents an ideal situation, in which no initial loss or redundancy occurs, such that at a library size of N less than b (the number of total possible peptides), there are N distinct peptides represented, for a coverage of N/b. Once the library size exceeds b, coverage stays at 1. Increasing library size always improves coverage until 100% coverage is reached. However, the added value gained from increasing library size decreases with increasing total size.

We therefore introduce relative efficiency of a library to measure the value returned for a library of a particular size and a specified scheme:

**Definition 2** (Relative efficiency). Relative efficiency is defined as the ratio of expected peptide diversity of a library relative to its overall number of oligonucleotides:

$$\begin{array}{ccc} R(N,k) & = & D(N,k)/N.\\ \sigma_{R}^{2} & = & \sigma_{D}^{2}/N^{2}. \end{array}$$

This makes relative efficiency a number between 0 and 1. A relative efficiency of 1 indicates that all peptide sequences in the library are unique and no sequence is found more than once. If the relative efficiency is close to 0 the level of redundant peptide sequences is high. A relative efficiency of 0.5 means that we expect half of all peptide sequences in a library to be valid and unique.

**Fig 2:**
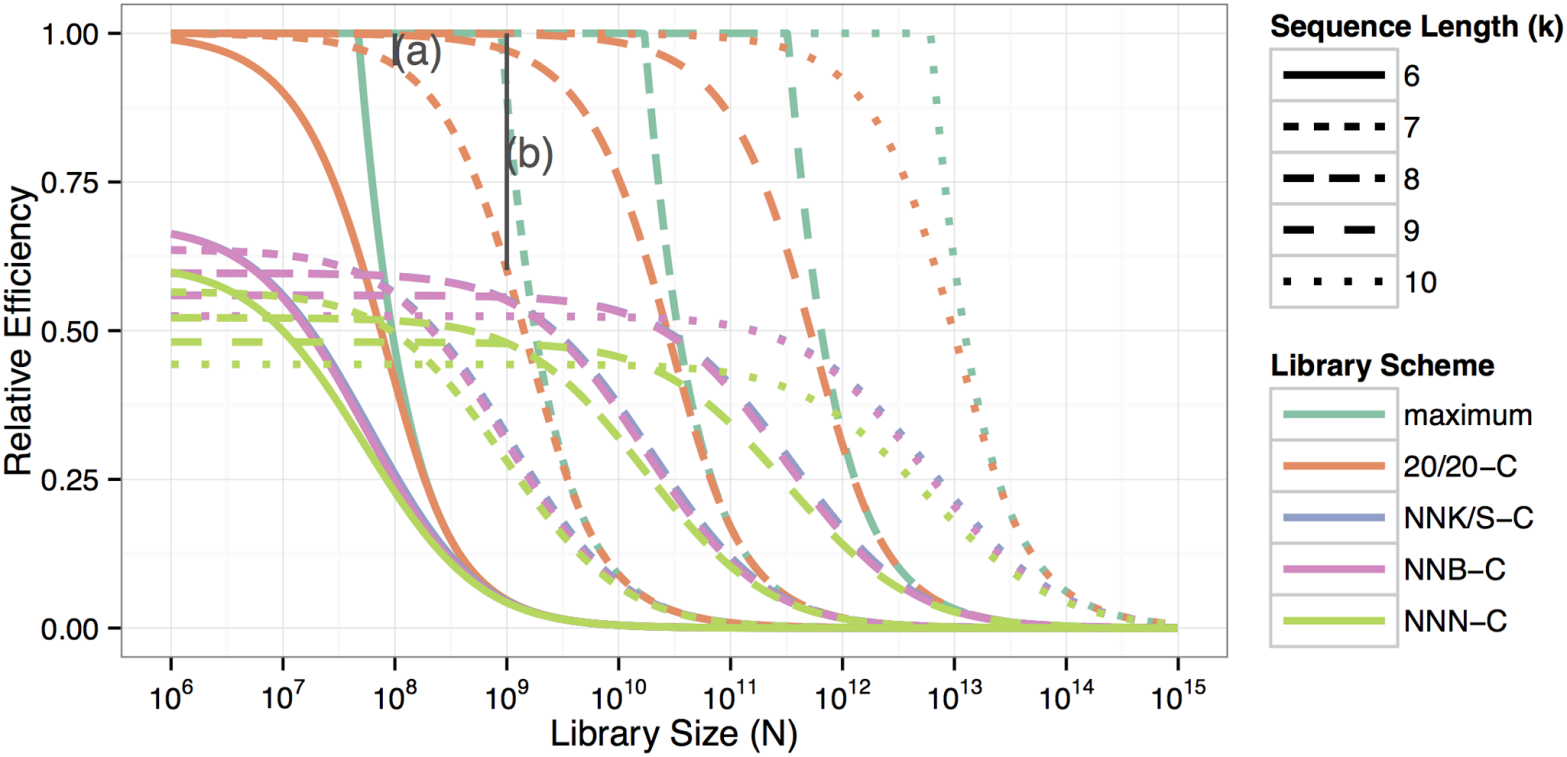
Overview of relative efficiency for k-peptide libraries (6 to 10) of sizes N from 10^6^ to 10^15^. Relative efficiency decreases with an increased number of oligonucleotides in the library and longer peptide sequencesdue to the larger initial loss.

Fig 2 gives an overview of relative efficiency of k-peptide libraries of various sizes. In contrast to an ideal situation or in a 20/20-C library, libraries encoded by NNK/S-C, NNB-C and NNN-C schemes suffer from an initial loss due to sequences containing aa class Z codons. This limits their maximal relative efficiency depending on encoding scheme and peptide length k. With increasing library size, relative efficiency decreases due to increasing effects of redundancy. In an ideal case, this drop only occurs when the library size reaches the maximal possible diversity for the given peptide length k. In practice, however, this loss becomes notable when a library reaches a size of about 1% of the maximal number of possible peptides.

Current AAV library sizes are in the order of 10^8^. Here, the loss due to redundancy makes up for less than 10% in heptapeptide 20/20 libraries (see (a) in Fig 2). As peptide libraries increase, the problem grows exponentially. In heptapeptide libraries of size 10^9^, the loss due to redundancy (see (b) in Fig 2) is 39.9%.

### Inclusion Probabilities

Full coverage—especially with longer peptide sequences—might be very difficult to achieve in practice. However, as Yuval Nov describes for saturation mutagenesis in protein evolution [46], it might not always be reasonable to aim for full coverage to ensure that the one ‘best’ sequence is included in a library (what is ‘best’ is always defined by the goals of a specific library selection, e.g. to identify the peptide that shows the strongest interaction with a protein). The reasoning behind this is simple: one would expect that there are in fact several highly similar peptides which perform similarly well. This assumption is supported by the fact that even in selections using libraries with incomplete coverage, we often observe an enrichment of several sequences that share common sequence motifs (e.g. [14, 29, 47]). With this in mind, it might be more reasonable, instead, to raise the question: “What diversity is necessary to find *at least one of* the best possible peptides?” To answer this, we first estimate the probability that the single best sequence is part of the library. In a next step we assess the probability that any related sequence from an appropriately specified sequence neighborhood around it is included.

The probability that a specific peptide sequence is present in a library depends on the overall size of the library and its scheme. Let p_i_ be the probability that peptide i is in the library, and $\sum_{i=1}^{t}p_{i}$ be the cumulative probability for the occurrence of any one of a group of t peptide sequences in the library. Define X to be the number of the specified t peptides that occur in a library of size N. The probability that at least one of the t peptides is in the library is then:

$$\begin{array}{ccc} & & P(X\geq 1)=1-P(X=0)=\\ & & \;=1-{\left(1-\sum_{i}^{t}p_{i}\right)}^{N}\approx 1-e^{-N\sum_{i}^{t}p_{i}}. \end{array}$$

The approximation is based on the same argument as Theorem 1 and holds for any reasonably large values of N.

**Fig 3:**
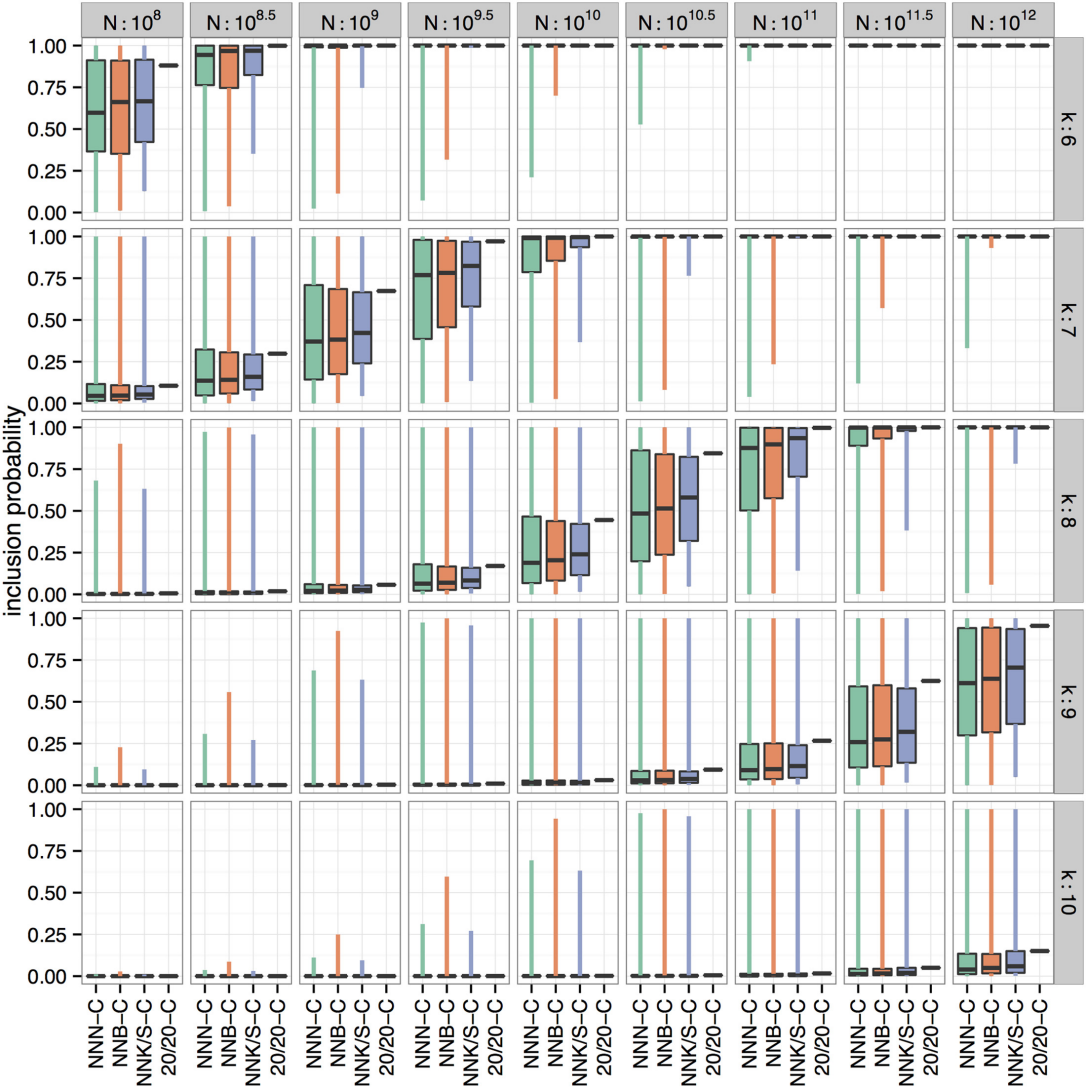
Overview of the inclusion probabilities for peptide sequences of lengths 6 to 10 (in rows) in libraries of sizes between 10^8^ to 10^12^ (in columns) for different encoding schemes (as side-by-side boxplots). The boxes contain the middle 50 percent of inclusion probabilities for all peptide sequences of length k in each of the schemes. The vertical lines extend to minimum and maximum of the inclusion probabilities. 20/20-C libraries do not have any variability in the inclusion probabilities, because all sequences are equally likely. NNN-C libraries generally show the largest variability (as seen in the extent of the boxes) in probabilities, followed by NNB-C and NNK/S-C. Simultaneously, median inclusion probabilities increase from NNN-C to 20/20-C libraries for all combinations of peptide lengths and library sizes.

The probability p_i_ of a peptide sequence to occur in a library depends on the number of codons of each of its amino acids. This number varies between library schemes, making an exact *a priori* assessment of the inclusion probability of the ‘best’ peptide sequence impossible except in the case of 20/20 libraries, in which each peptide sequence occurs with equal probability. In all other library schemes, the probability of sequences to be included in the library is highly variable (see also [20]). Fig 3 gives an overview of just how much the probability of including the ‘best’ peptide sequence varies in each encoding scheme with different library sizes. Side-by side boxplots show the inclusion probabilities of all peptide sequences for each peptide length k from 6 to 10 and library sizes N between 10^8^ and 10^12^. The colored boxes contain the middle 50% of all possible peptide sequences. 20/20-C libraries (shown in pink) do not have any variability associated with the inclusion probability, indicating that all peptide sequences have an equal chance to be part of the library. NNN-C libraries have the largest variability associated with them, while NNK/S-C libraries have the smallest (after 20/20-C libraries).

The high variability introduced by schemes with varying codons per amino acid ratios causes libraries to be biased towards peptides with a high number of possible encodings at the cost of rare ones. This makes the chance of success in selections strongly dependent on the question, if the *a priori* unknown “best” peptide has many possible encodings or not. Therefore, the inclusion probability for some peptides is maximal in biased schemes like NNN-C and for peptides with high number of encodings inclusion probabilities exceed those achievable with 20/20-C encoding (see S4 Text and S5 Table). However, for about 75% of all possible peptides the highest inclusion probability is reached when an unbiased coding scheme like 20/20-C is used (see Fig 3).

### Neighborhoods

To determine if at least one of the best possible peptides (or a “top” peptide) is included in a given library, we have to define first what a *top* peptide is. For that we use a rather restrictive definition: a top peptide is any peptide that differs from the best possible peptide s in up to one (first degree neighborhood) or up to two (second degree neighborhood) amino acid positions which are conservatively exchanged. To objectively define conservative exchanges we employ the BLOSUM80 matrix [48], which provides log-odds scores for the chance to observe a substitution of one amino acid for another. Only exchanges with a positive BLOSUM80 score were considered in determining neighborhoods of top peptides. Further, exchanges to stop codons and cysteines were defined here to lead to invalid sequences. In general, a neighborhood of degree d includes all sequences that differ in at most d amino acids from peptide s. It is obvious, that a degree d-neighborhood of s includes s itself as well as all sequences of neighborhoods of a lower degree than s.

Neighborhoods and their sizes depend on the individual peptide sequence. Therefore, we cannot give a single inclusion probability, but we rather have to cite a range of probabilities for including top peptides. To set the boundaries of this range, we consider a best and a worst case scenario under all encoding schemes. In the worst case scenario, the top sequence consists of amino acids with only a single codon each (minimizing the probability to be part of the library) along with the smallest possible number of viable exchanges (minimizing the size of the top peptide neighborhood). Analogously, the top sequence in the best case scenario is one that consists of amino acids with a maximum number of codons in the encoding scheme (maximizing the probability to be found in the library) combined with the largest possible number of viable exchanges (maximizing the size of the top peptide neighborhood).

**Fig 4:**
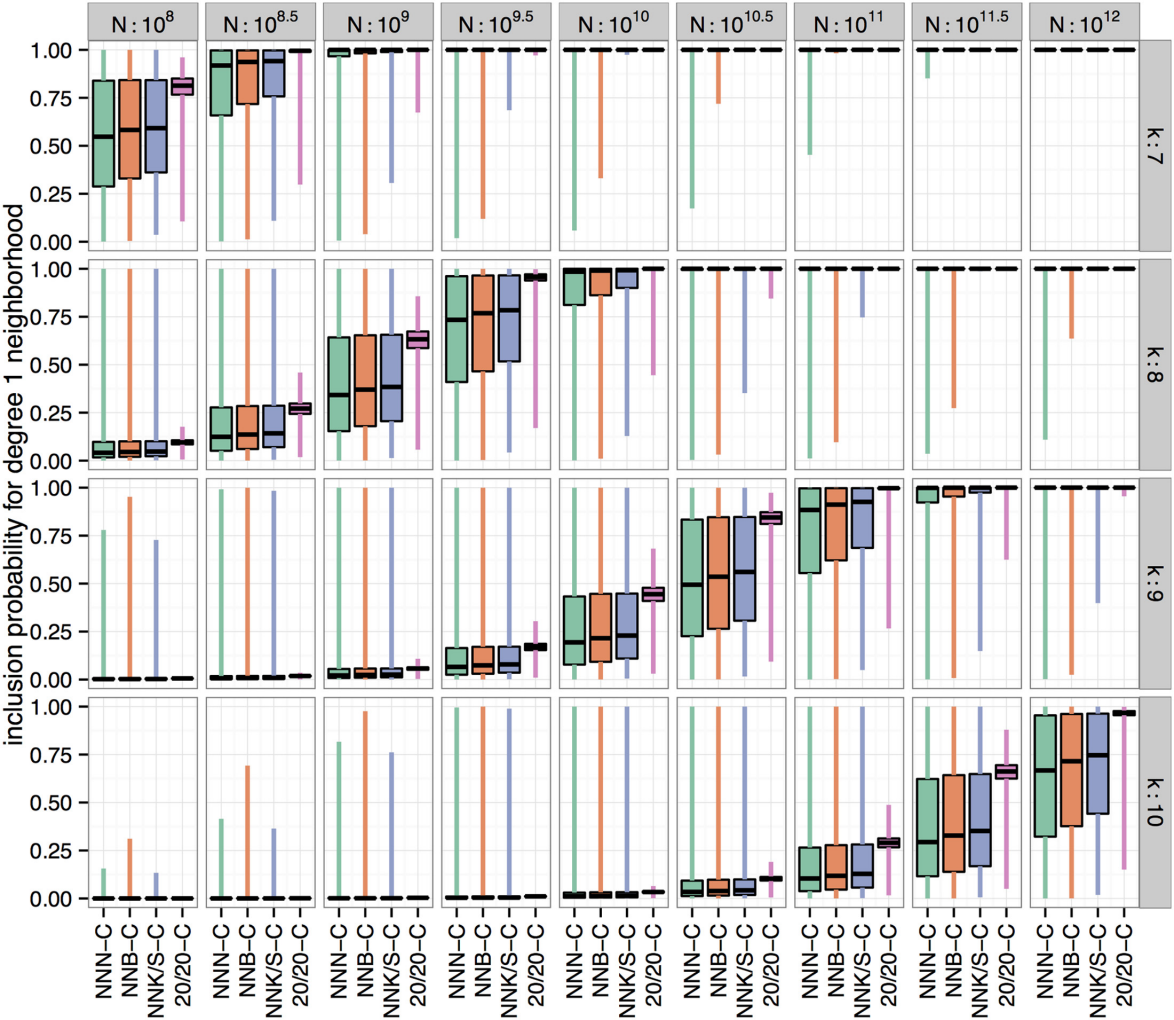
Side-by-side boxplots of the probabilities that at least one of the sequences belonging to the first degree neighborhood of the best sequence is included in libraries of different sizes (columns) and different lengths of peptides (rows). Best and worst case probabilities depend on the number of encodings for a sequence and the exchangeability of the amino acids it consists of.

Fig 4 gives an overview of the probabilities of including one of the sequences in the first degree neighborhood of the best peptide sequence of length k = 7. For an NNK/S-C library of size one billion (N = 10^9^), we have a minimum chance of about 30% (worst case scenario) that one of the sequences of the first degree neighborhood around the best heptapeptide sequence is included. This chance increases to close to 100% for more than 75% of all peptide sequences. Taking a one degree neighborhood of peptide sequences into account has roughly the same effect on inclusion probabilities as considering sequences of a shorter length (k-1) or using a library of more than ten times the size. Note that a switch from best sequence to first degree neighborhoods of the best sequence does not change the effect that library schemes have on inclusion probabilities except for libraries, which show a higher variability in inclusion probabilities.

For individual sequences we can calculate the probability of including any of its d-degree neighbors (for d = 1, 2) based on the BLOSUM80 matrix, see S5 Table for an example.

In particular for longer peptide sequences, higher degree neighbors might play a significant role in the analysis of results. While theoretically feasible, practically neighborhoods of higher order can only be derived -due to computational limitations- for a limited set of peptide sequences rather than the whole library.

## Discussion

Peptide library selection is a powerful technology used in a wide variety of biological systems. For an optimum exploitation of this technique, it is necessary to understand the properties of the peptide libraries. Currently however, the possibilities to functionally describe a peptide library are rather limited. Several publications exist that focus on mathematical descriptions of saturation mutagenesis libraries used in protein evolution ([16, 43, 49, 50], among others). While saturation mutagenesis and peptide library display are similar in many aspects, they differ in the fact that in the first generally only low numbers of isolated positions are randomized while in the second often long randomized peptides are used. This causes differences in the techniques available for randomization and, especially, in the number of possible sequences and thereby in the mathematical complexity. Therefore, researchers designing new peptide libraries have to choose key parameters like peptide length, encoding scheme, and target diversity without a possibility to adequately quantify the effects of their decisions. Available qualifiers like functional diversity and number of bacterial colonies offer some degree of information, but are unsuited to compare the properties of different libraries in detail. We present a mathematical framework to determine the number of distinct peptides and to calculate the estimated coverage and relative efficiency. These properties are implemented in the web-based tool PeLiCa (http://www.pelica.org) and enable researchers to quantify and compare their libraries in far greater detail, which in particular allows for a more informed planning of new libraries and projects. Researchers can use the preset library schemes in PeLiCa as well as define new ones. The core of our approach is to classify peptides according to the redundancy of their encodings first, and then use these peptide classes to regard individual peptide sequences in a second step. This two-step procedure reduces the complexity of the problem sufficiently, making a mathematical assessment of complete libraries analytically feasible. The sheer size of most peptide libraries causes alternative approaches to fail. Direct simulation, for instance, is impossible to implement on standard machines due to the limitations of main memory and disk space. Even if these hurdles were taken by more sophisticated simulation strategies, the process would be too slow to be of practical use. For very small library sizes a simulation study is discussed in S3 Text, which shows the accuracy of the theoretical framework in practice (S3 and S4 Tables). For somewhat larger library sizes, the validity of our approach was successfully confirmed by direct comparison with GLUE-IT [37]. GLUE-IT determines protein diversity and coverage for small libraries of individual proteins with mutations in up to 6, in general non-consecutive, amino acid positions (“saturation mutagenesis generated protein libraries”). Though the biological setting is different from the peptide libraries discussed here, GLUE-IT can be used to analyse a limited set of peptide libraries with very short randomised inserts (k = 1 to 6; Cysteines defined as valid; comparison in S6 and S7 Tables). In reverse, our approach and website can also be used to investigate saturation mutagenesis libraries.

In this publication, we limit our examples to peptides of 6 to 10 amino acids in length, as shorter peptides are rarely used and the use of longer peptides—even for very large current libraries (N up to 2 × 10^10^)—results in an expected coverage close to zero. The relative efficiency in these cases stays close to its possible maximum defined by peptide length and encoding scheme (Figs 1 and 2). The losses in efficiency are strongly dominated by the initial loss and a relative efficiency R (defined in definition 2) captures the ratio of the number of viable codons and all codons in the scheme.

The most fundamental information about a peptide library is the number of encoded peptides. However, determining this value is difficult, as it is not only influenced by the number of clones generated in library production, but also by other factors. Our framework is able to determine a value for the peptide diversity from the number of bacterial colonies by figuring in statistical and encoding effects as well as prominent biological factors (stop codons and cysteines). The negative influence of these factors has already been discussed in the past (e.g. [19–23, 27, 45, 51–53]), however, our system now allows a quantification of their effects. Based on peptide diversity we calculate the relative efficiency and the expected library coverage. The standard deviations for all three measures are negligibly small for reasonably sized libraries. For example an NNK-C heptapeptide library of size 100 Million has a peptide diversity of 5.6 × 10^7^ ± 9.6 × 10^3^, an expected coverage of 6.3% ± 1.1 × 10^-5^% and a relative efficiency of 56.3% ± 9.6 × 10^-5^%.

Information on coverage is important to put libraries and selection results in perspective (see also [37]). In the above example (NNK-C library; k = 7; N = 10^8^) only about 6% of all possible sequences are covered. Therefore, it is not likely that the most prominent sequence selected from this library is in fact the best possible heptapeptide. Besides that, two identical selections using independent libraries might result in identification of two completely different sets of selected peptides. This situation can be improved by either increasing the library size, which is often restricted by technical limitations, or by changing to a more favourable library design (see also [16, 21, 45, 46]). Of the encoding schemes investigated here, 20/20-C is the most beneficial regarding expected coverage and relative efficiency, as it avoids the initial loss and suffers less from redundancy effects. It also prevents the bias for amino acids with a high number of codons shown by other schemes. As generating 20/20 libraries with the trimer technique is still rather expensive, the majority of current applications uses libraries with other encoding schemes. However, there are alternative techniques to trimer to reach a ratio of one codon per amino acid, like the MAX randomization [20], the “small-intelligent libraries” [22] and the ProxiMAX randomization [23]. Of these only ProxiMAX is suited to produce the longer randomized sequences needed for most peptide library applications [23].

**Fig 5:**
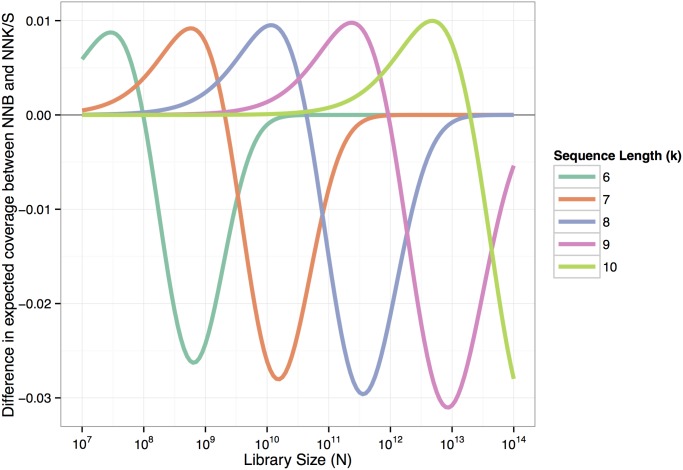
Difference in expected coverage between NNB and NNK/S libraries (with cysteines). Initially, NNB libraries have a slight advantage in expected coverage over NNK/S libraries. Once a coverage of about 50% is reached, this pattern reverses and NNK/S libraries have a highere expected coverage. For very large libraries the difference in coverage is again, approaching zero (when libraries under both schemes have a coverage of almost 100%).

When comparing different library schemes regarding expected coverage and relative efficiency, NNK/S-C and NNB-C are very similar and preferable to NNN-C (see Figs 1 and 2). NNK/S-C has a slight advantage over NNB-C in peptide diversity, expected coverage, and relative efficiency. If cysteines are considered as viable, however, NNB encoding has a minor advantage over NNK/S for libraries with a low expected coverage (Fig 5). The initial advantage in expected coverage of NNB over NNK/S is due to the smaller initial loss of NNB: out of 48 codons, 47 are valid (corresponding to a 97.8% of valid codons), leading to a loss of 1—(47/48)^k^, whereas NNK/S has 31 valid codons out of 32 (corresponding to a 96.9% of valid codons), leading to a (slightly) higher loss of 1—(31/32)^k^. When peptide sequences including cysteine are also considered as invalid (in NNK/S-C and NNB-C schemes), the advantage of the initial loss disappears, because then an equal percentage of 93.75% of all codons are valid under either scheme.

NNK and NNS are mathematically identical but differ biologically due to different codon preferences of the host organisms. In *E. coli* and especially in *S. cerevisiae*, codon usage suggests that NNK may generally be the better option [19], while in human cells NNS codons are preferred. Another important design factor is the peptide length, as an elongation by one amino acid increases the number of possible peptides by a factor of 19 (20/20-C) to 23 (NNN with cysteines). When planning a new library, one should therefore consider the biological demands on peptide length on the one hand and the achievable coverage on the other. For all discussed encodings except 20/20 or 20/20-C, peptide length does not only influence the coverage but also the absolute number of viable peptides, as the chance that disruptive codons (stop codons and cysteines if relevant in the system) are included, increases with length. In fact, there is an optimal length that maximizes peptide diversity and relative efficiency for any given library size N (Fig 2). For example, for a non-20/20-C library of size N = 100 Million a peptide length of k = 8 is optimal in the sense, that its relative efficiency is larger than for libraries of peptide lengths 7 or 9. Therefore peptide diversity of a library of 8-peptides is also maximal.

Even extremely large libraries rarely exceed N = 10^10^, using peptides longer than 9 to 10 amino acids therefore leads to a reduced peptide diversity in non-20/20-C libraries. In the case of an NNK-C library of 10 billion sequences about 40% less viable peptides are contained if a length of 18 amino acids is used instead of the optimal 9.

A high coverage is not always feasible due to limited library size and biological restraints on peptide length. Therefore, the chances that the “best” peptide is included in the library are often slim. However, peptides whose sequences are close to ideal may exist and perform similarly well [46]. By calculating the chance that at least one such peptide is contained, it is possible to better evaluate if a specific library is likely to produce high performing peptides. This chance depends on the used encoding scheme and the sequence of the “best” peptide. As this sequence is unknown beforehand, we define a best and worst case and determine a probability range for different library designs (see Fig 4). The degree of variability is by far smallest for 20/20-C libraries indicating that such libraries should show the most reliable performance over different selections. With the worst case scenario as the most relevant qualifier, 20/20-C is again the best scheme followed by NNK/S-C, NNB-C and NNN-C, with differences spanning several orders of magnitude. About 5 × 10^9^ sequences are needed in a heptapeptide 20/20-C library for a 99.5% chance that even in the worst case at least one top peptide from the first degree neighborhood is part of the library. With NNN-C about 2000-fold more (10^12^) sequences are necessary.

In summary, our mathematical framework and its implementation at the web-interface PeLiCa offer evaluation parameters that allow an in-depth analysis of peptide libraries. This promotes a better understanding of library dynamics and enables a more informed design process. With the help of this mathematical framework libraries can be optimised directly for the requirements of the experiment and for the technical feasibility in a given setting. Therefore, our work contributes to improved peptide libraries which, in turn, will impact the success of viral and phage display systems in a multitude of scientific applications.

## Supporting Information

S1 Text Proof of Theorem 1. (TEX)

S2 Text Approximation Error in Theorem 1. (TEX)

S3 Text Simulation Results. (TEX)

S4 Text Examples for inclusion probability. (TEX)

S5 Text Validation of results based on established software sources. (TEX)

S1 Table NNN-C Library Scheme. (XLS)

S2 Table NNB-C Library Scheme. (XLS)

S3 Table Observed library diversity compared to expected library diversity for different library sizes N under various library schemes. (XLS)

S4 Table Variances of peptide diversity for different library sizes N under four library schemes. (XLS)

S5 Table Inclusion probabilities of specific heptapeptide sequences (degree 0) and first and second degree neighborhoods (degree 1 and degree 2) under different library schemes in a library of size 100 Million DNA Sequences. (XLS)

S6 Table PeLiCa vs GLUE-IT comparison of peptide diversity/expected number of distinct amino acid variants in hexapeptides. (XLS)

S7 Table PeLiCa vs GLUE-IT comparison of Coverage/Completeness of hexapeptides under different library schemes and library sizes. (XLS)

S1 Fig Simulation results: 100 libraries of size 105 were sampled from each of the library schemes NNN, NNK/S, NNB, and 20/20, and the number of unique peptide sequences in each library was determined. (TIF)
