# Supplementary figures and images for: Biomathematical Description of Synthetic Peptide Libraries
Source: PLoS One. 2015 Jun 4;10(6):e0129200. doi: 10.1371/journal.pone.0129200 (PMC4456392; doi:10.1371/journal.pone.0129200)

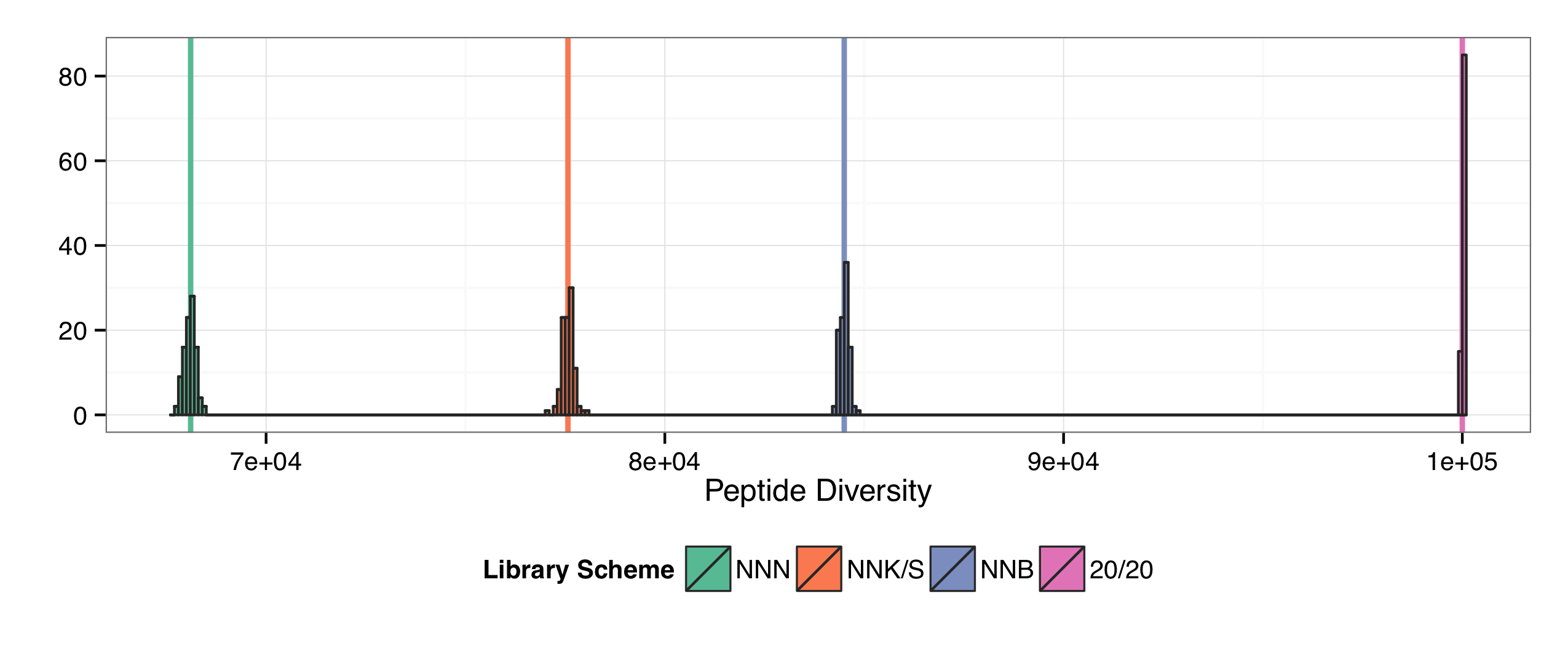

Supplement: S1 Fig — (TIF) [file pone.0129200.s013.tif]
